# Care pathways for longstanding eating disorders must offer paths to recovery, not managed decline

**DOI:** 10.1192/bjb.2023.38

**Published:** 2024-06

**Authors:** James Downs

**Affiliations:** Patient Representative, Faculty of Eating Disorders, Royal College of Psychiatrists, UK

**Keywords:** Anorexia nervosa, eating disorders not otherwise specified, bulimia nervosa, patients, nosology

## Abstract

Eating disorders are historically underserved in healthcare, but are increasingly prevalent and recognised for their high costs regarding mortality, quality of life and the economy. Those with longstanding eating disorders are commonly labelled ‘severe and enduring’ (SEED), which has been challenged for its conceptual vagueness and potential to discourage patients. Attempts to define individuals from this cohort as having ‘terminal’ illness have also gained traction in recent years. This paper is grounded in lived/living experience and relevant research. It challenges the logical coherence and utility of SEED, arguing that the word ‘enduring’ unhelpfully situates intractability of longstanding illness within patients themselves and the nature of their illness. This risks a sense of inevitability and overlooks the important role of contextual factors such as lacking resources and insufficient evidence for withholding active treatment. Recommendations suggest approaches to dismantling unhelpful binaries between early intervention and intensive support, recovery and decline.

## Eating disorders: a field in need of sustenance

Historically under-served across research,^1^ medical training^2^ and service provision,^3^ eating disorders are now more widely acknowledged as a growing priority in healthcare.^4^ Encompassing diagnoses such as bulimia nervosa, binge eating disorder, other specified feeding and eating disorders, and anorexia nervosa, eating disorders can carry significant psychological and physiological risks,^5^ have high rates of mortality^6^ and create significant economic and personal costs.^7^ Epidemiological data is limited,^8^ and increasing rates of hospital admission in England^9^ have been exacerbated by evidence that the COVID-19 pandemic provided a ‘perfect storm’ for eating disorders to develop.^10^ These factors together should sharpen the minds of policy makers in their efforts to increase the resources provided for high-quality, accessible eating disorder services, particularly in light of a recent survey of specialist services in England and Scotland indicating that services receive less than a fifth of what they require.^11^

Initiatives to improve the quality of care and alleviate the high costs of eating disorders have tended to focus on early intervention and the prevention of illness, and consequently have prioritised younger age groups.^12^ These efforts must be continued and expanded. Missing, however, are clear guidelines for patients like myself, with more longstanding and severe eating disorders, who may have missed the boat for early intervention but nevertheless require a substantive evidence base for treatment options that may work and be accessible for them.^13^

## Emerging care pathways for longstanding illnesses

For too many people, the course of illness with eating disorders can span years.^14^ The lack of access to services both presently and historically (e.g. specialist adult services only existed in Wales after 2008^15^) mean that people may grow into illness, rather than out of it. This can result in a more difficult to treat disorder, sometimes accompanied by mistaken attitudes among healthcare professionals that frame patients as unwilling to embrace change.^16^

This cohort of patients was first identified as having a ‘severe and enduring eating disorder’ (SEED) in 2015.^17^ Since then, what is without doubt an accurate way to describe a clinical presentation has been used (and experienced by patients)^18^ as a ‘label’ or diagnostic category in itself. The proposed characteristics of such patients include longer duration of illness, psychological features such as decreased motivation to change, and an emphasis on those with low body mass index (producing a subcategory of ‘severe and enduring anorexia nervosa’) on the grounds of the physiological risk that this poses.^19^

Currently, however, there is no official sub-diagnostic criteria for SEED within diagnostic classification manuals,^20^ let alone adequate research or clinical consensus regarding what care patients who are very unwell over a long period of time may best respond to. Practice on the ground shows that many specialist eating disorder services recognise the need to provide ongoing support or tailored treatment pathways for when first-line psychological treatments may not be suitable, yet this is not consistent in approach or distribution across the UK. For instance, some pathways focus more on quality-of-life goals, and exclude treatment targets such as eating disorder psychopathology and weight status. More recent research has emphasised the importance of including an orientation toward change-focused treatments and recovery in conventional diagnostic terms.^21^ These efforts are valuable, in particular for their emphasis on the views of patients, and findings that support the need to coproduce services that embrace a shared language around this subject.

Although those with severe eating disorders who may not have been in contact with services for a long time might rightfully be ambivalent about the outcomes of treatment the support they may have to change, we must be wary of the creation of an unhelpful binary between ongoing support and active treatment, where ongoing support is not ultimately geared toward recovery, and instead could be perceived as writing off the possibility of recovery rather than bridging the significant gaps that can exist in moving toward it. This is reflected in the views of patients about the terminology of SEED being negatively focused, lacking in hope and interpreted in a way that results in less support from healthcare instead of more.^20^

## Personal concerns and conceptual problems

In my own experience of eating disorder, those treating me told me I would likely never recover fully in my lifetime, even only a few years into my illness. Perhaps in a flawed attempt to inspire me to recovery through fear, I was reminded that the prognosis would become even bleaker the longer I remained unwell – all of this while still being only a teenager. To this day, I still have not recovered, after nearly 20 years of illness, although I have made some progress. I wonder to what extent those professionals were correct, but mostly about their reasoning. Is it that eating disorders become unresponsive to treatment after time, or that they do not respond so well to the type and intensity of treatment that is offered? Have I not recovered because my illness is inherently more intractable over time by its very nature, or because I have only had 18 months of treatment in nearly two decades of severe illness? What role did having to wait over 6 years from diagnosis to specialist treatment play?

I wonder also about the possibility that we are creating a problem here that does not exist. By saying there is the existence of this thing called ‘SEED’, we may bring into being a specific problem within patients that suggests a categorically different approach is needed, rather than maintaining that these patients sit within the array of presentations of eating disorder, of which some can be described as more severe and longstanding than others. In doing so, we may obfuscate the most pressing and enduring problem of all in relation to the existence of longstanding and severe illness: the lack of access to evidence-based care for patients and their families at the time and intensity that is required to support recovery.

The most basic idea that more severe and chronic forms of illness would require more intense and sustained treatment must also apply for patients with eating disorders, as would be expected for any health condition. Considering the limited evidence base within eating disorder research,^22^ in particular in relation to diverse presentations and the role of comorbidity in treatment, it would be a mistake to suggest focusing on the characteristics of patients and nosological change without first building the evidence base for how effective existing treatments are when delivered within the context of accessible and adequately resourced services. There is evidence that patients who receive an increased intensity of treatment achieve better outcomes compared with treatment as usual, irrespective of length of illness.^23^

To situate within patients the many and obvious problems underpinning the inability to adequately provide enough care of sufficient quality via existing means would be highly premature, not least before exhausting all solutions that may be of offered first by a vast expansion of research and investment in services for eating disorders. From understanding aetiology and complex interactions across bodily systems, to interrogating the best practice in service design, to creating treatment that flexes with diversity of clinical presentation, there is so much we do not yet know. Increasing our knowledge base and innovation in treatment must be prioritised before writing off existing diagnostic categories of eating disorders as incapable of encompassing the varying degrees of severity, chronicity and treatment needs of patients.

## A diagnosis of despair

Contrary to these ambitions, it has been suggested that patients with a SEED who have not responded to conventional recovery-focused treatment as hoped should be offered less of it, not more. A prominent example is Gaudiani et al's proposal, on the basis of a very small sample of patients, of the category ‘terminal anorexia nervosa’.^24^ Although the characteristics of these patients are unestablished, the proposed solution being offered is to cease offering treatment for eating disorders and only offer palliative care for its effects, with the rationale being that treatment would cause more harm than good because of the ‘intractable’ nature of their illness.

The idea of eating disorders as terminal is perhaps a natural product of the use of the terminology of SEED and, in particular, the word enduring, from the Latin, ‘*durus*’ (hard). The etymology of ‘*in*’ and ‘*durus*’, or hardening, portrays eating disorders as progressive illnesses by their very nature, with symptoms hardening and continuing to harden over time. To describe someone as having an enduring illness is to say their condition is continuing to get more established and predicts that it will do so. This is distinct from saying it has endured to this point, and to this point only. Labelling longstanding eating disorders as enduring in and of themselves risks relegating the importance of intervention, and its possible effectiveness. It can also overlook the vital role of context in determining whether or not a condition has endured in the first place.

Instead, one could externalise the persistence of eating disorders from the patient themselves, or the nature of their condition, to contextual factors such as the persistent lack of care that might have been available, or how patients may have had to endure treatments that are inadequately targeted, researched or resourced to meet their specific needs. Chronic inadequacy in services must be an equal or greater focus of concern in understanding the reasons why eating disorders can persist and worsen over time. It is premature to claim that we have all the tools available to treat patients with SEEDs, and to problematise the nature of their illness rather than the basis of our current knowledge and resources to help.

As a patient, it also strikes me as antithetical to the principles and aspirations of medical care to put efforts into providing an evidence base to predict with certainty who will not respond to treatment, and who is incapable of recovery. I am unaware of psychopathology itself being terminal, and conflating this with existing ethical and practice guidelines around terminal illness and failure of the body's vitality seems like a dangerous generalisation with potentially harmful consequences, including from the narratives this generates.

At a very simple level, the availability of an additional diagnosis of ‘terminal’ eating disorder means that is something that can be argued for, or requested by patients. Reading descriptions of the characteristics by which these patients could be defined,^23^ I cannot help but reflect how I would have been a fitting candidate, and could perhaps have requested this care pathway myself rather than be engaged (sometimes forcefully) in treatment. I have gone on to live many years of what I experience as a life worth living, and understand my failure to recover as meaning I have not been able to reliably access the treatment and conditions that may bring that recovery about. When I have accessed more intense treatment, I have progressed. As such, patients with SEEDs like myself do not have ‘hardening’ illnesses that, by their nature, are untreatable irrespective of the treatment offered. We have, instead, often experienced a system that is unable to treat us. Defining patients like me in such negative ways can terminate our opportunities for recovery by closing doors to treatment and denying us the hope that we can get better. The harms of this and the possibility of enduring hope over enduring illness have been written about with eloquence and power by other patient advocates whose experiences must be listened to.^25^

## The role of hope in creating recovery

Hope has an important role in recovery from eating disorder,^26^ and patients with longstanding illnesses have highlighted hope as a key ingredient to the care they would like to receive.^20^ In my own experience, hopeful messages were largely absent from my interactions with healthcare. Instead, I was inspired to recover through fear of adverse consequences, or denied treatment as a hopeless case who would not be able to get better because I had not already. This provided a self-fulfilling prophecy as I felt either so overwhelmed by fear that I was not able to take action, or like my hope was so diminished that there would be no point in trying to change. At other times, I have experienced hope as a sole strategy in the face of resignation that nothing else could work, that all we could do is hope for the best rather than take action.

There have been many times when I have not been able to have hope that recovery is possible. When I had not been able to recover by the means I had tried, I saw this as predictive that any efforts to recover in future were going to be unsuccessful. This kind of predictive thinking with insufficient evidence could be described as a cognitive error, proven inaccurate by my ability to make significant steps in recovery through engaging in further, more intensive treatment. The fact people have not recovered via the means they have available does not exclude the possibility that other means of recovery under different conditions could lead to a different outcome – the evidence is simply not there. Treatment for eating disorders that is concerned with psychopathology could also benefit from addressing false predictions about recovery for which there is no definitive evidence, rather than reinforcing them.

There is much evidence that people can recover after many years of illness, including studies showing that illness duration is not a good predictor of outcomes, including recovery.^27^ This research directly contradicts the theoretical basis of SEED, serving as a reminder for clinicians and researchers that the current lack of evidence-based treatments for longstanding eating disorders does not equate to them being untreatable. The potential for recovery should also be communicated to patients, without pretending that there are not significant gaps in our current knowledge of what might work for them. Services also need license to try unconventional treatments, which may not be in current National Institute for Health and Care Excellence guidelines^28^ but may show promise with further research, such as oxytocin^29^ and ketamine.^30^ Services also need to have the resources to evaluate the efficacy of innovative practice, to contribute to an evolved evidence base for longstanding eating disorders.

Being creative is at the heart of knowledge generation, and recovery on an individual level can be described as a creative endeavour rather than a reversion to some prior state that may be a distant memory for some patients with longstanding illness. A focus on creating a life ‘worth living for’ is central to care pathways proposed for SEED,^20^ and I am struck in my own experience by how I was never asked what kind of life I wanted to have without my illness, only ever told that I needed to remove it from the picture. Focusing on quality of life does not have to mean writing off the idea of recovery being possible. Rather, it can give patients a taste of what to recover for, and a direction toward the support they need to do so.

## Active recovery versus decline: a false binary

This space in-between active recovery and managed or unmanaged decline is a neglected one. As a patient, I have witnessed a binary in how services are organised for patients like me. At one end, you have to be ready and able to engage in change-based eating disorder treatment (on a timescale and in a format determined by services), and if you are not then you can feel blamed when treatment does not work for you, or are labelled as ‘unengaged’ when you may have felt like it was actually the service that struggled to engage with you. On the other hand, too many people with longstanding illnesses are unable to access care at all, or are defined as terminal, which can close doors to recovery.

Between these poles, we need services where patients can build alliance with a service and therapeutic relationships as a fundamental basis for recovery,^31^ rather than be forced into change prematurely by services they may not trust. We need careful management of the physiological effects of eating disorders over time without assuming people can never recover psychologically from their illness. We need honesty that, more often than not, it is not the case that patients with eating disorders are untreatable – it is that services are unable to treat them.^32^ Although these grey areas may be harder to negotiate than the black and white extremes, this is the reality of the complexity of longstanding eating disorders, and services must be designed accordingly. Directly or obliquely, all types of support must be oriented toward recovery, be that timely access to change-focused therapy or encouragement in laying the foundation for it.

Services must also be equipped to be able to comprehend the nature of many longstanding eating disorder presentations as entangled with co-occurring conditions.^33^ Access to specialist assessment and reassessment where needed can help unlock alternative treatments and ways of treating more holistically, taking into account the whole picture of someone's illness.^34^ In my case, it was nearly two decades after the onset of my eating disorder that I was diagnosed with autism spectrum disorder, attention-deficit hyperactivity disorder and Ehlers–Danlos syndrome, despite all of these having involvement in how I developed and maintained my illness. Having this knowledge has shaped my treatment positively, unlocking opportunities for recovery that were previously overshadowed by a sense of hopelessness that all options were exhausted, when in fact they were not.

Intimately linked to the idea of hope is the role of agency. In my experience, believing in my ability to enact change was a prerequisite for trying in the first place, as was knowing that the support I needed to do so could be trusted and depended upon for however long I might need it. Similarly, having a voice in my care has been key. I have often felt like I have been told what is wrong with me and what to do. Although this may have come from a place of clinicians wanting (or needing) to help me, it resulted in a loss of agency, which has been described by others with eating disorders^35^ and their carers.^36^ It has greatly helped me to be asked what sense I make of my own experiences, and to think about how this can inform a shared understanding in alliance with those caring for me.

## Recommendations

Creating the treatment, research basis and service design that patients with longstanding eating disorders need requires significant resources. Increased investment will save lives, which are too often lost as a result of lack of access to treatment.^37^ A more hopeful future for those with longstanding eating disorders will be made more likely by embracing the following recommendations:

- Collaboration is needed with patients and their supporters in creating shared understandings of illness, treatment and recovery. This can start with asking patients about their experiences and preferences in how they are described or labelled. Replacing the label ‘SEED’ with more neutral descriptors such as ‘longstanding’ would provide a more open basis for this.
- Services must provide options for patients with longstanding illness to establish the prerequisites for conventional recovery-focused treatment. These care pathways must be co-produced meaningfully with those who require them.
- Dedicated resources for longstanding eating disorders must be radically increased. These include, but are not limited to, the following: service funding; training and skills; human resources, including patient involvement; and research.
- Culture change is required to move away from binaries such as that between early intervention and longstanding illness, or change-focused treatment and managed decline. Efforts to solve the problems posed by longstanding eating disorders should not prioritise seeking evidence for who will not respond to treatment. We must, at all times, be guided by the protection of life and the possibility of recovery.

## About the author

**James Downs** is a patient representative with the Faculty of Eating Disorders, Royal College of Psychiatrists, UK.
